# EnRank: An Ensemble Method to Detect Pulmonary Hypertension Biomarkers Based on Feature Selection and Machine Learning Models

**DOI:** 10.3389/fgene.2021.636429

**Published:** 2021-04-27

**Authors:** Xiangju Liu, Yu Zhang, Chunli Fu, Ruochi Zhang, Fengfeng Zhou

**Affiliations:** ^1^Department of Geriatric Medicine & Shandong Key Laboratory Cardiovascular Proteomics, Qilu Hospital of Shandong University, Jinan, China; ^2^College of Computer Science and Technology, and Key Laboratory of Symbolic Computation and Knowledge Engineering of Ministry of Education, Jilin University, Changchun, China

**Keywords:** EnRank, ensemble feature selection, filter, pulmonary hypertension, biomarker detection

## Abstract

Pulmonary hypertension (PH) is a common disease that affects the normal functioning of the human pulmonary arteries. The peripheral blood mononuclear cells (PMBCs) served as an ideal source for a minimally invasive disease diagnosis. This study hypothesized that the transcriptional fluctuations in the PMBCs exposed to the PH arteries may stably reflect the disease. However, the dimension of a human transcriptome is much higher than the number of samples in all the existing datasets. So, an ensemble feature selection algorithm, EnRank, was proposed to integrate the ranking information of four popular feature selection algorithms, i.e., T-test (Ttest), Chi-squared test (Chi2), ridge regression (Ridge), and Least Absolute Shrinkage and Selection Operator (Lasso). Our results suggested that the EnRank-detected biomarkers provided useful information from these four feature selection algorithms and achieved very good prediction accuracy in predicting the PH patients. Many of the EnRank-detected biomarkers were also supported by the literature.

## Introduction

Pulmonary hypertension (PH) shows the symptom of high blood pressure in the lung arteries which impedes the delivery of blood from the heart to the lungs (Mandras et al., 2020). PH is diagnosed by at least 20 mmHg (millimeter of mercury) of the rest-state mean pulmonary arterial pressure (mPAP) and the right-sided heart catheterization (Simonneau et al., 2019). Although PH may be caused by various factors, the PH patients painfully suffer from shortness of breath and increased mortality (Mandras et al., 2020). As many as, 10% of people over age 65 are affected by PH, and more than half of them develop heart failure (Hoeper et al., 2016). Detection of novel transcriptomic biomarkers may facilitate the understanding of the PH molecular mechanisms and serve as candidates for investigation and prognosis of the disease (Jardim and Souza, 2015; Swaminathan et al., 2015).

The high throughput DNA sequencing technology generates the expression levels of all human protein-coding and non-coding genes (Jandl et al., 2019; Tzimas et al., 2019), and machine learning methods rely on this data (Stephens et al., 2015; Mirza et al., 2019). The sequenced samples may be lesion tissues, e.g., the endothelial cells or the small remodeled arteries (Jandl et al., 2019; Tzimas et al., 2019). The peripheral blood mononuclear cells (PBMCs) serve ideally as the targets for the transcriptome sequencing because it is less invasive than use of lesion tissue (Tzouvelekis et al., 2018).

Transcriptome based disease prediction is often limited by sufficient sampling toward detection of disease features. This is mostly caused by the high cost of sequencing, a transcriptome and the difficulty in recruiting a cohort of individuals with and without the disease (Diao and Vidyashankar, 2013). Building a prediction model using all features may lead to overfitting and loss of applicability to a non-training data set (Schinkel et al., 2019). This problem is addressed by an algorithm for selecting a subset of the features in building the disease prediction model (Schinkel et al., 2019; Shi et al., 2019; McCabe et al., 2020).

There are two main categories of feature selection algorithms, filter and wrapper (Ye et al., 2017). A filter feature selection algorithm evaluates the association of each feature with a class label and then ranks features based on the significance of this association (Hall and Smith, 1999). These filter algorithms are commonly used for detection of biomarkers since their time complexity is linear. However, the filters ignore the inter-feature correlations and cannot detect the subset of low ranked features with good prediction performance (Ye et al., 2017). A wrapper feature selection algorithm heuristically generates the feature subsets and evaluates the prediction performance of a given feature subset using a user defined classifier (Das, 2001). The wrappers usually have higher time complexities than the filters and tend to deliver the feature subsets with better prediction performance than the filters (Ge et al., 2016).

This study proposes an ensemble feature selection algorithm, EnRank, to take advantage of both filters and wrappers. The main idea of EnRank is to integrate the ranks of multiple feature selection algorithms and verify that the final feature subset is efficient for use in a prediction model. A comprehensive evaluation was carried out to test which classifier achieved the best prediction performance. Our experimental data suggested that different feature selection algorithms may contribute complementary information to each other and the orchestration of the features selected by these algorithms are efficient for use in a predictive model.

## Materials and Methods

### Collection of Data

This study used the transcriptome dataset GSE33463 of pulmonary hypertension patients and controls (Cheadle et al., 2012). The gene expression levels were profiled from the PBMCs of the recruited participants on the microarray platform GPL6947 (Illumina HumanHT-12 V3.0 expression beadchip). Each sample had 49,576 transcriptomic features, and the feature annotations were retrieved from the platform definition file.

This dataset consisted of 140 samples in total. There were 30 idiopathic pulmonary arterial hypertension (PAH) patients, 19 patients with systemic sclerosis (SSc) without pulmonary hypertension, 42 scleroderma-associated PAH patients, and eight patients with SSc complicated by interstitial lung diseases and pulmonary hypertension. The remaining 41 samples were non-disease controls. This study investigated the binary classification problem between the 99 patients (positive samples) and 41 non-disease controls (negative samples).

### Feature Selection Algorithms

Feature selection algorithms were used to find the biomarkers with the best disease detection performance. Each sample had 49,576 transcriptomic features, and the overall dataset had 140 samples in total. A classification model may have a large chance of overfitting for this “large *p* small *n*” situation (Keel et al., 2019; Ren et al., 2020). A feature selection algorithm may be used to find a subset of features for building an accurate and stable classification model. This would also make the model easier to be interpreted along with better performance during the training step. The following four feature selection algorithms were utilized to find a good subset of features.

The Chi-squared test (Chi2) helps to test the relationships or dependence between two variables. Chi2 may be used to remove the features without dependency on the class labels (Xiao et al., 2020). In other words, these removed features will have a small contribution to any classification model.

The T-test (Ttest) is widely used to evaluate the statistical significance (*p* value) of the null hypothesis that a feature of the positive samples has the same normal distribution as that of the negative samples (Govindan et al., 2019; Soh et al., 2020). A feature with the value of *p* < 0.05 is typically considered a candidate for differential expression between the positive and negative samples. In addition, a feature with a lower *p* value is considered to have increased power for binary classification.

The ridge regression (Ridge) evaluates a subset of features for their connections with the class labels (Gao et al., 2020; Xu et al., 2020). Ridge provides a model-based trade-off between the fitting and complexity of the features by adding the L2 regularization to the regression model.

The Least Absolute Shrinkage and Selection Operator (Lasso) algorithm adds the L1 regularization to the regression model along with a penalty value for number of features (Deshpande et al., 2019). So, Lasso tends to select a small subset of features and weights them for building a robust regression model.

### Binary Classification Methods

The models for predicting disease were trained using five binary classifiers.

Logistic regression (LR) is a statistics model using a logistic function to model a binary classification problem (Cuadrado-Godia et al., 2019; Khandezamin et al., 2020). The logistic model calculates the log-odds for the class label by a linear combination of one or multiple predictors.

Support vector machine (SVM) is a supervised machine learning algorithm originally designed for binary classification (Jin et al., 2019; Wang et al., 2019). SVM searches for a hyperplane to separate two classes of samples with the maximal margins. It enriches the feature space through a kernel function to quantify the inter-sample similarities.

A simple algorithm K Nearest Neighbor (KNN) is a popular supervised machine learning framework for both classification and regression tasks (Wang et al., 2020; Yuan et al., 2020). KNN determines the class label of a query sample through the majority voting strategy of the KNNs of the query sample.

Decision tree (DT) uses a tree structure to solve the classification problem (Prieto-Gonzalez et al., 2020). Each node except for the leaves in a DT classifier exerts a feature evaluation, and the evaluation result determines which sub-branch of this current node to follow. DT is a simple and easy-to-interpret classifier.

The adaptive boosting tree (AdaBoost) is an integrated machine learning technique (Qiao and Xie, 2019; Dou et al., 2020). The weight of a sample will be increased if this sample leads to a misclassified base classifier. Each iteration will add new base classifiers. The final goal is to find a strong classifier with sufficiently small error rate.

### Performance Evaluation Metrics

The supervised machine learning algorithms were evaluated by the following performance metrics. These metrics are essential to measure a prediction model from different aspects. This study used specificity (Sp), sensitivity (Sn), accuracy (Acc), and the area under the receiver operating characteristics curve (AUC). The number of correctly predicted positive samples was defined as the true positive (TP) and that of the incorrectly predicted positives was the false negative (FN). The true negative (TN) and the false positive (TP) defined the numbers of correctly and incorrectly predicted negative samples, respectively.

The overall accuracy is calculated as the number of all the correct predictions divided by the total number of samples in the dataset. That is to say, Acc = (TP + TN)/(TP + FN + TN + FP). The value of Acc is between 0.0 and 1.0. The two metrics Sp and Sn describe the ratios of correctly predicted negative and positive samples, respectively. So Sp = TN/(TN + FP) and Sn = TP/(TP + FN). Both metrics are between 0.0 and 1.0. A larger value of the three metrics Acc/Sp/Sn suggests a better prediction performance. The Matthews’ Correlation Coefficient (MCC; Matthews, 1975) was introduced by the biochemist Brian W. Matthews in 1975 and MCC is generally regarded as a balanced measurement which can be used even if the classes are of very different sizes. The metric AUC is a parameter independent metric for the prediction model and shows a trade-off between Sp and Sn (Shao et al., 2020).

### The Proposed Feature Ranking Algorithm, EnRank

This study proposed the ensemble feature selection algorithm, EnRank, by calculating the weighted ranks of the four feature selection algorithms, i.e., Ttest, Chi2, Ridge, and Lasso. The two filter algorithms Ttest and Chi2 rank the features by their individual association values of *p* with the class labels. The two linear fitting algorithms Ridge and Lasso rank the features based on the absolute values of the fitted model’s coefficients. The values of the feature ranks start from 1, i.e., the best ranked feature has the rank 1. Each feature selection algorithm selects top-ranked pTopK = 100 features for further screening.

The proposed algorithm EnRank defines a weight *Aim_i_* for each feature selection algorithm, where *i*∈{Ttest, Chi2, Ridge, Lasso}. The pTopK features selected by each algorithm were loaded into the five classification algorithms, i.e., LR, SVM, KNN, DT, and AdaBoost. The stratified 5-fold cross validation (S5FCV) strategy was used to calculate the metric AUC, and each feature selection algorithm received five AUC values. This study aimed to find a feature subset with stably high AUC values for five classification algorithms, and defined *Aim_i_* = *Avg_i_*/*Var_i_*, where *Avg_i_* and *Var_i_* were the averaged value and variance of the five AUC values of the feature selection algorithm *I*, respectively.

Finally, EnRank generated an integrated rank for each feature *f*. To avoid the case of very low ranking features, the rank of feature *Rank_i_*(*f*) was redefined as the penalization rank pPenaltyRank = 1,000, if *Rank_i_*(*f*) > pTopK. The integrated rank EnRank(*f*) = Average(*Rank_i_*(*f*) × *Aim_i_*) was defined as the EnRank metric, where the function Average() is the averaged value, and *i*∈{Ttest, Chi2, Ridge, Lasso}.

Then, any filter-based feature selection frameworks, e.g., the incremental feature selection (IFS), may be used to find the best subset of top-ranked features generated by EnRank.

### Workflow of This Study

**Figure 1:**
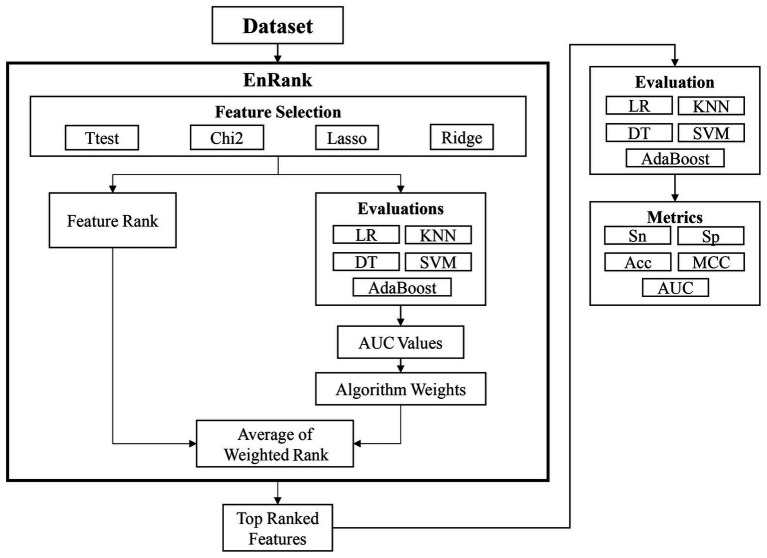
Workflow of this study. The proposed algorithm EnRank integrates the ranks of both feature selection algorithms and classification algorithms. The finally generated feature subset is further evaluated by five different classification algorithms.

This study proposed an ensemble feature selection algorithm, EnRank, by integrating the feature ranks from different algorithms (Figure 1). The experimental data in the following section suggested that different feature selection algorithms performed differently, and it is necessary to integrate the ranking information calculated by different feature selection algorithms.

## Results and Discussion

### Comparison of the Feature Ranks by Ttest and Chi2

Table 1 illustrated the top-ranked 10 features delivered by the two filter algorithms Ttest and Chi2. Firstly, the statistical significance *p* values of the two algorithms Ttest and Chi2 were different to each other. The minimum *p* value of Ttest was 2.83e-19 while Chi2 only calculated the minimum *p* value 4.08e-4 for its null hypothesis. Actually, even the rank-100 feature ILMN_1698668 by Ttest had value of *p* = 2.28e-12, which was much smaller than the minimum value of *p* = 4.08e-4 of the algorithm Chi2.

**Table 1:** The top-10 features ranked by Ttest and Chi2. The two columns “Ttest” and “Chi2” gave the names of the ranked features.

Ttest	Ttest-*p* value	Rank	Chi2	Chi2-*p* value
ILMN_1812970	2.83E-19	1	ILMN_1806023	4.08E-04
ILMN_1875248	9.71E-19	2	ILMN_1656011	8.59E-04
ILMN_1704335	2.28E-18	3	ILMN_1702691	1.48E-03
ILMN_1794233	6.85E-18	4	ILMN_2367126	2.42E-03
ILMN_1765725	1.06E-17	5	ILMN_2339955	3.21E-03
ILMN_1758687	1.83E-17	6	ILMN_1815527	3.44E-03
ILMN_1687526	5.60E-17	7	**ILMN_1789074**	5.21E-03
ILMN_1767168	7.40E-17	8	ILMN_1782305	5.49E-03
ILMN_2159384	1.38E-16	9	ILMN_2088437	8.62E-03
**ILMN_1789074**	5.47E-16	10	ILMN_1751607	9.14E-03

Column “Rank” provided the rank values. “Ttest-*p* value” and “Chi2-*p* value” provided the statistical *p* values as calculated by the two algorithms Ttest and Chi2. The feature was highlighted in bold if it was among the top-ranked 10 features of both algorithms.

And there was only one feature ILMN_1789074 shared among the top-ranked 10 features by Ttest and Chi2. The *p* value for the Ttest null hypothesis was 5.47e-16 for feature ILMN_1789074 (Ttest rank 10), while Chi2 recommended ILMN_1789074 as the rank 7 feature with *p* value 5.21e-3.

So, the statistical tests Ttest and Chi2 generated significantly different *p* values for the features, and we had to integrate the features by their rank values.

### Comparison of the Feature Ranks by Ridge and Lasso

Only six out of the top-10 ranked features by the absolute values of their model coefficients were shared by the two algorithms Ridge and Lasso (Table 2). This study assumed that both positive and negative correlations of the features with the class labels were important, and the absolute values of the model correlation coefficients of these features were used to rank the features in descending order. The feature ILMN_1697499 was the best ranked feature by Ridge, but it was not even within the top-10 ranked features by Lasso. Actually, the feature ILMN_1697499 was ranked 26 by Lasso. And the best ranked feature ILMN_1678859 by Lasso was only the ninth ranked feature by Ridge.

**Table 2:** The top-10 features ranked by the model coefficients of the regression models Ridge and Lasso.

Ridge	RidgeCoef	Rank	Lasso	LassoCoef
ILMN_1697499	0.0671	1	**ILMN_1678859**	0.1663
**ILMN_2165753**	0.0563	2	**ILMN_1781236**	0.1589
**ILMN_1807491**	0.0484	3	ILMN_2058782	0.1362
**ILMN_1781236**	0.0435	4	**ILMN_2083066**	0.1307
**ILMN_1806023**	0.0435	5	**ILMN_1807491**	0.1269
**ILMN_2083066**	0.0428	6	**ILMN_1806023**	0.1142
ILMN_1721113	0.0425	7	ILMN_1801216	0.1120
ILMN_2229649	0.0408	8	ILMN_1822671	0.1078
**ILMN_1678859**	0.0398	9	ILMN_1789074	0.1077
ILMN_2323933	0.0395	10	**ILMN_2165753**	0.1072

The two columns “Ridge” and “Lasso” identified the top-10 ranked features. Column “Rank” provided the rank values. The two columns “RidgeCoef” and “LassoCoef” gave the absolute values of the model coefficients calculated by the two algorithms Ridge and Lasso. The features were highlighted in bold if they were among the top-ranked 10 features of both algorithms.

**Figure 2:**
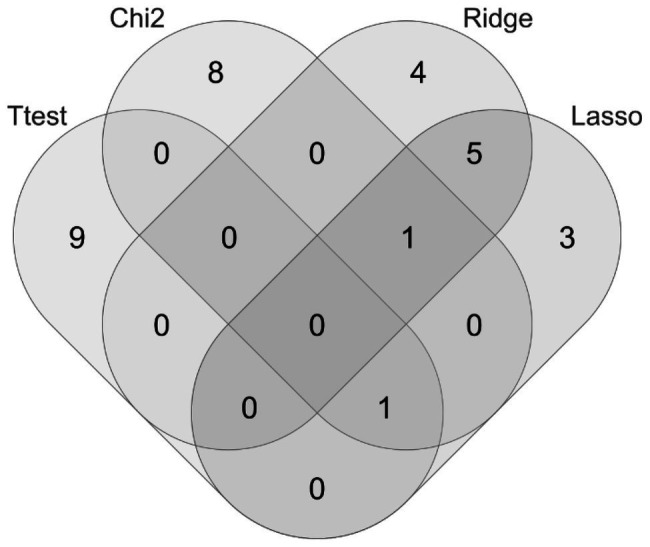
Venn diagram of the top-10 features ranked by the four feature selection algorithms. The feature selection algorithms were T-test (Ttest), Chi-squared test (Chi2), Least Absolute Shrinkage and Selection Operator (Lasso), and ridge regression (Ridge).

Venn diagram (Figure 2) shows that very few features were shared by these four feature selection algorithms, i.e., Ttest, Chi2, Lasso, and Ridge, except between Lasso and Ridge.

**Figure 3:**
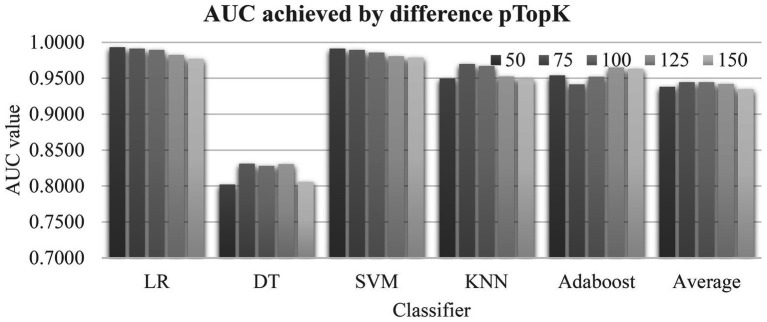
Evaluation of the parameter pTopK of EnRank. The horizontal axis listed the five classifiers and the averaged area under the receiver operating characteristics curve (AUC) values by pTopK value. The five values, 50/75/100/125/150, are from EnRank.

The data in Tables 1, 2 suggested that the top-ranked features by the four algorithms Ttest, Chi2, Ridge, and Lasso described the class correlations of the features from different aspects. Figure 3 evaluated different value choices of the parameter pTopK. Both pTopK = 75 and 100 achieved the best averaged AUC = 0.9446. In order to introduce more feature diversity, this study focused on the four lists of top-ranked pTopK = 100 features by the above four algorithms, and their union consisted of 269 features.

### Evaluation of the Four Feature Selection Algorithms

**Figure 4:**
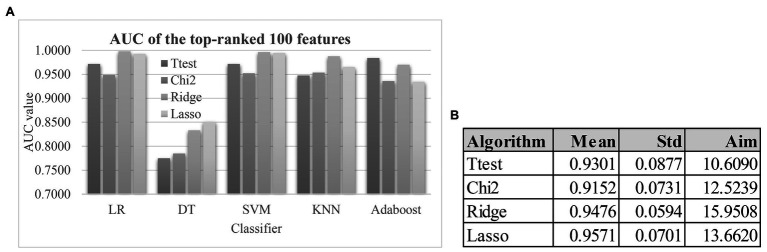
The model performances and the weights of the four feature selection algorithms. **(A)** The AUC values of the top-100 features ranked by the four feature ranking algorithms using the five classification algorithms. Each of the four feature ranking algorithms Ttest, Chi2, Ridge, and Lasso selected the top-ranked 100 features. The AUC values of the feature lists were calculated by the stratified 5-fold cross validation (S5FCV) strategy of the five popular classification algorithms. **(B)** Calculation of the algorithm weight “Aim” for each of the four feature selection algorithms. The columns “Mean” and “Std” were the mean values and the SDs of the five classification algorithms. And the column “Aim” was defined as Mean/Std.

Figure 4A demonstrated that the classification algorithm DT had low performance on all four feature lists. And the other four classification algorithms achieved at least 0.9000 in the metric AUC for all four feature lists. Although the Lasso-selected 100 features achieved the best mean AUC value 0.9571 by the five classification algorithms, its SD 0.0701 was larger than that (0.0594) of another algorithm Ridge. So the Lasso’s Aim 13.6620 was slightly larger than that (15.9508) of Ridge, as shown in Figure 4B. The filter Ttest was assigned the Aim 10.6090 due to its largest SD 0.0877.

### Distribution of the Calculated EnRank Metrics

**Figure 5:**
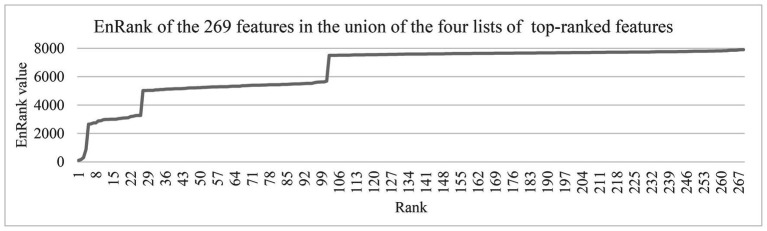
The EnRank metrics of the 269 features in the union of the four lists of top-100 ranked features. The horizontal axis gave the feature ranks ordered by the EnRank metric, and the vertical axis gave the EnRank metrics of the top-ranked 269 features. These features were among the union of the top-100 ranked features recommended by the four algorithms, Ttest, Chi2, Ridge, and Lasso.

The ranking metric EnRank was defined in the above subsection “The proposed feature ranking algorithm, EnRank.” EnRank used the EnRank metrics to rank the features in ascending order, and the features were roughly separated into four groups, as shown in Figure 5. The EnRank metrics of the ordered features were within these four ranges, i.e., [1, 1,000], [2,500, 3,300], [5,000, 5,700], and [7,500, 7,900]. The experimental data suggested that these four groups of features consisted of features recommended by four, three, two, and one feature selection algorithms, respectively. That is to say, a feature recommended by four feature selection algorithms was not penalized by the penalization rank pPenaltyRank, and algorithm aims were between 10 and 16. Such a feature had an EnRank smaller than 1,000. So the metric EnRank reasonably described how each feature was ranked by multiple feature selection algorithms.

### Literature Supportive of the EnRank-Detected 50 Biomarkers

The metric Literature Support (LR) of a feature was defined by the number of PubMed (Fiorini et al., 2017) publications matching the gene symbol of this feature and the key word “pulmonary disease” in both title and abstract. The query term was “term={}[tiab] AND pulmonary disease [tiab],” and the queried date was November 16, 2020. The cumulative LR (CLR) of the top-k ranked features was defined as the sum of the LR values of these *k* features.

**Figure 6:**
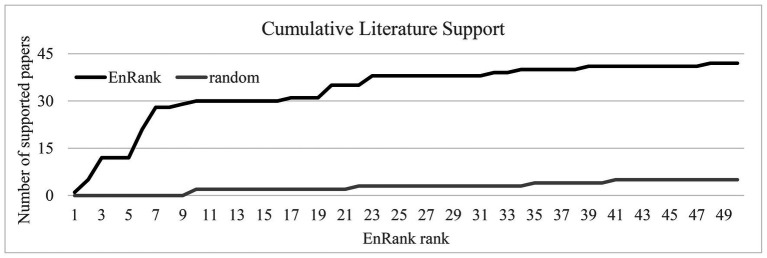
Evaluation of the cumulative literature support LR (CLR) of the top-50 EnRank-ranked features. The horizontal axis gave the EnRank-recommended ranks and the vertical axis shows the metric CLR.

In order to compare with the biomarkers selected by EnRank, we randomly selected the same number of genes among the remaining genes as a control group, and then compared the metrics CLR and LR in the two groups. Figure 6 illustrated that the EnRank-detected top-ranked features were investigated for their roles in pulmonary diseases many more times than the randomly-chosen features. The randomly-chosen features were supported by at most two PubMed publications, and only four out of the 50 randomly-selected features had literature support. And the EnRank-detected top-ranked 50 features were more significantly supported by the scientific literature. Some features were supported by as many as nine PubMed publications, and 14 out of the 50 features had literature support. So the EnRank-detected features were consistently supported by the literature.

### Model Evaluation Based on the EnRank-Detected Biomarkers

**Figure 7:**
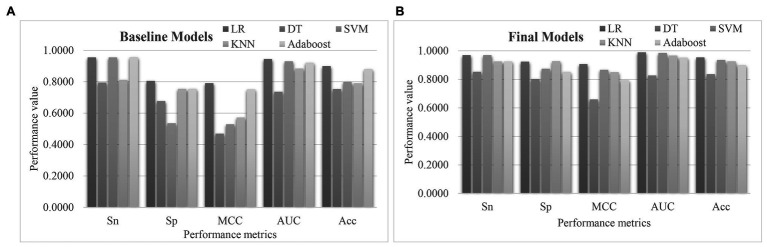
Performance comparison of the five classification algorithms. The S5FCV strategy was used to train the five classification algorithms using **(A)** all the transcriptomic features, and **(B)** the 50 EnRank-detected biomarkers. The horizontal axis gave the performance metrics sensitivity (Sn), specificity (Sp), Matthews’ Correlation Coefficient (MCC), AUC, and accuracy (Acc). The vertical axis gave the values of these performance metrics.

A comparative study was carried out to evaluate whether the proposed algorithm EnRank recommended features with good prediction performance of pulmonary hypertension (Figure 7). The baseline models in Figure 7A showed that the classifier DT achieved the worst PH prediction accuracy (Acc = 0.7545), while the classifier LR achieved the best Acc = 0.9000. SVM achieved the same Sn = 0.9560 as LR, but much worse Sp = 0.5361 than that (Sp = 0.8056) of LR. So, it is necessary to find a subset of biomarker features with a better PH prediction accuracy.

Figure 7B showed that the 50 EnRank-detected biomarkers improved the prediction accuracies of all five classification algorithms. The largest improvement in Acc (0.1364) was achieved for both SVM and KNN. The classification algorithm LR achieved the best Acc = 0.9545 again using the 50 EnRank-detected biomarkers. The parameter-independent metric AUC = 0.9894 of LR was also the best among the five classification algorithms.

So this study delivered a PH prediction model using the 50 EnRank-detected biomarkers and the LR classification algorithm.

### Further Validation of the Proposed PH Biomarkers

**Figure 8:**
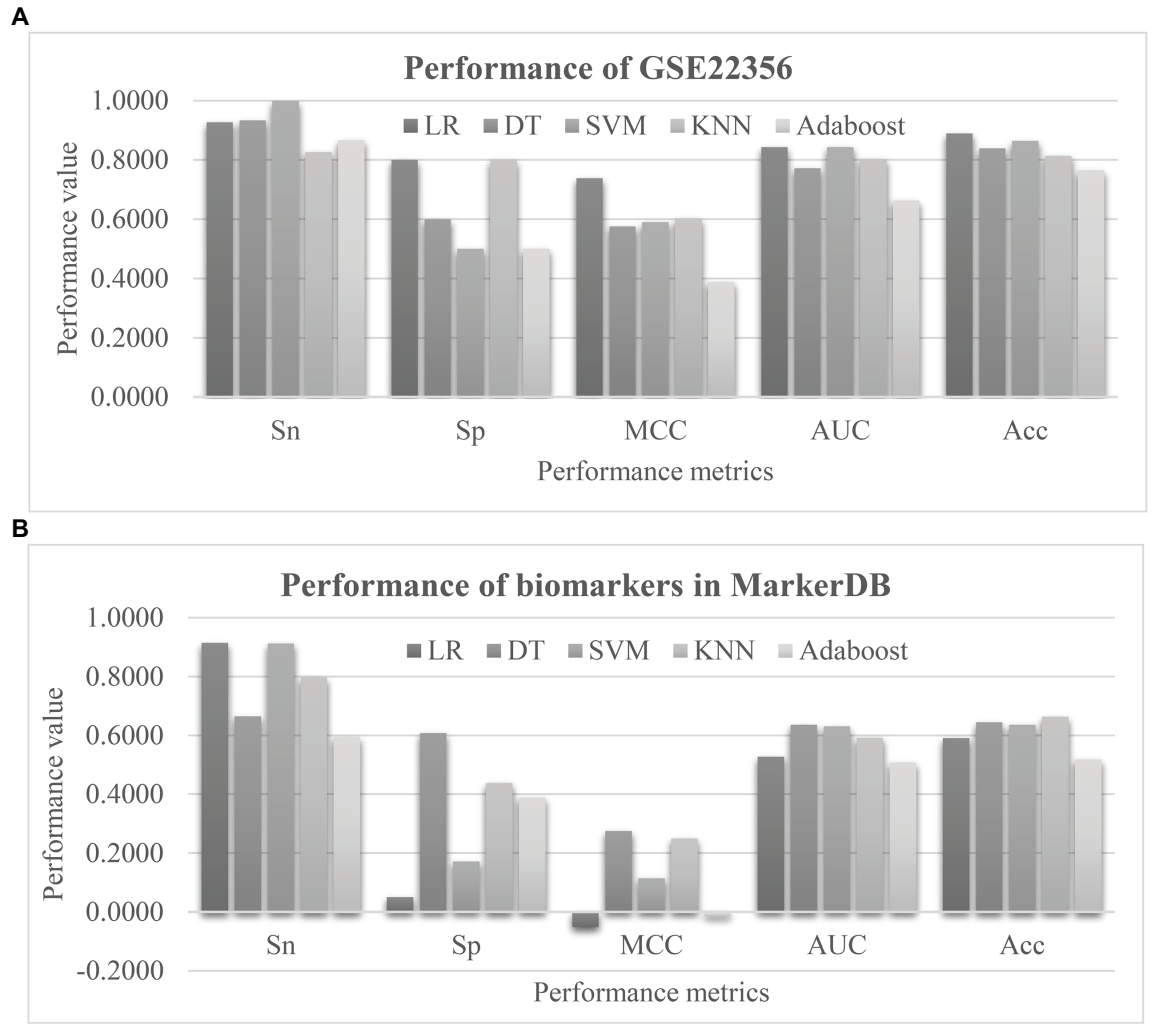
Evaluation of the pulmonary hypertension (PH) detection model. **(A)** Validation of the 50 EnRank-recommended PH biomarkers in the dataset GSE22356. **(B)** Performances of the existing PH biomarkers. The horizontal axis listed the performance metrics Sn/Sp/MCC/ROC/Acc and the five classifiers were given as data series. The vertical axis gave the values of the performance metrics.

Firstly, the proposed PH biomarkers were validated using an independent dataset GSE22356 (Risbano et al., 2010) from the Gene Expression Omnibus (GEO) database (Clough and Barrett, 2016). This dataset consists of 38 PBMC samples profiled by the Affymetrix Human Genome U133 Plus 2.0 Array (GPL570), and investigates the altered immune phenotypes of the scleroderma-associated pulmonary hypertension (Risbano et al., 2010). The normal controls were assumed as the negative samples, and the other samples were regarded as the positive ones. The 50 EnRank-recommended features matched 236 features through 36 unique genes in the independent dataset. The same settings of training and evaluation as EnRank were used. Figure 8A showed that four of the five classifiers achieved AUC values at least 0.8000. The classifier LR achieved the largest AUC = 0.8433, and the largest Acc = 0.8893. Considering that this independent dataset was profiled using a different transcriptome platform than our original dataset GSE33463, the independent validation results supported the robustness of the EnRank-recommended PH biomarkers.

We searched the literature database PubMed using the keywords “pulmonary hypertension” and “biomarker” in the titles, and only 41 publications were detected. Most of them focused on the protein (Wu et al., 2020), vocal (Sara et al., 2020), and imaging (Jivraj et al., 2017; Jose et al., 2020) data. So we collected the PH marker genes from the recently updated database MarkerDB (Wishart et al., 2021). Three unique genes were annotated as the PH biomarkers, including Bone Morphogenetic Protein Receptor Type 2 (BMPR2), Activin A Receptor Like Type 1 (ACVRL1), and Endoglin (ENG). Four features were associated with these three genes. The prediction performances of these four biomarker features were shown in Figure 8B. Unfortunately, no classifiers showed larger than 0.7000 in either AUC or Acc using these biomarkers. This should be due to that the existing biomarkers were screened for their individual associations with the phenotype PH, and their combined PH prediction performances were not investigated in the existing studies.

### Further Evaluation of Other Feature Selection Combinations

**Figure 9:**
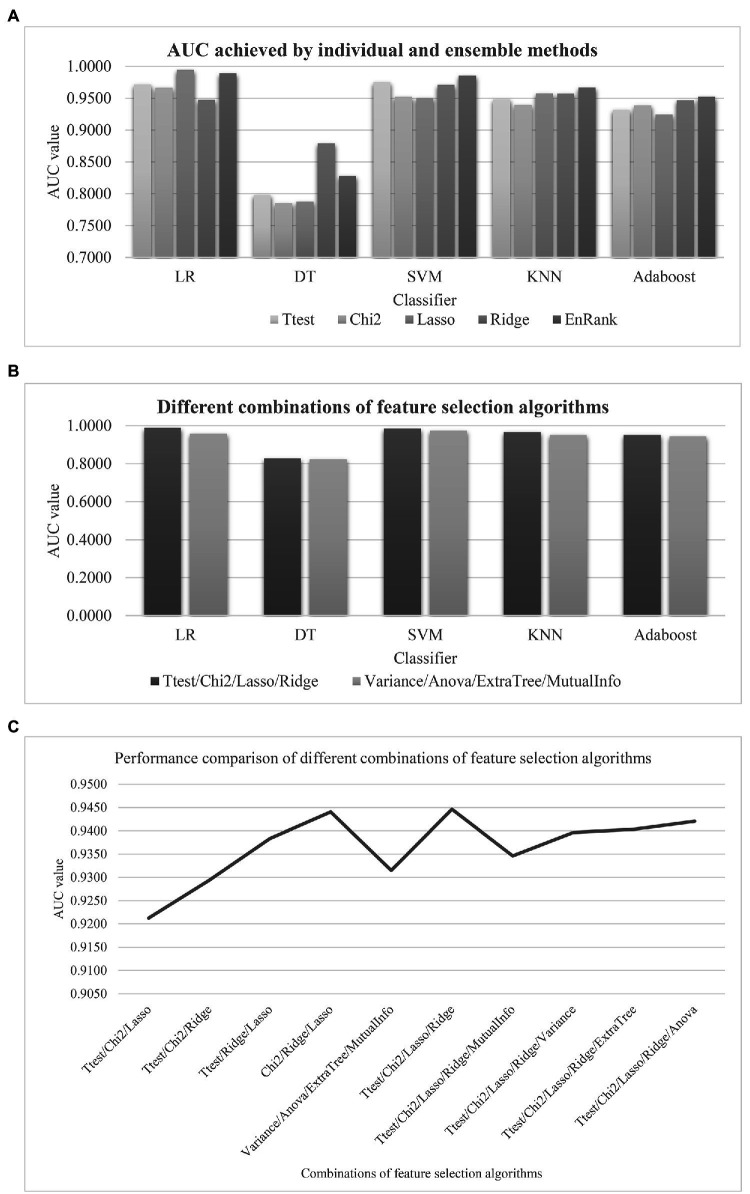
Comparison of EnRank with the other feature selection algorithms and their combinations. **(A)** Evaluation of EnRank and its individual feature selection algorithms. **(B)** Two groups of feature selection algorithms were integrated by EnRank. The original version of EnRank was “Ttest/Chi2/Lasso/Ridge,” and the new version was “Variance/Anova/ExtraTree/MutualInfo.” The horizontal axis listed the classifiers and the vertical axis gave the AUC values of the evaluated models. The ensembled algorithm EnRank and its four individual feature selection algorithms. **(C)** The AUC values of different combinations of feature selection algorithms averaged over the five classifiers logistic regression (LR)/decision tree (DT)/support vector machine (SVM)/k nearest neighbor (KNN)/adaptive boosting tree (AdaBoost). The horizontal axis listed the algorithm combinations, and the vertical axis gave the AUC values.

The proposed algorithm EnRank is a feature selection framework that may integrate the ranking data of multiple feature selection algorithms. The above sections integrated four feature selection algorithms, i.e., Ttest, Chi2, Ridge, and Lasso. Figure 9A evaluated the proposed ensembled algorithm EnRank and its four individual feature selection algorithms using the same training and testing settings. The parameter-independent metric AUC was used to compare the performances of the feature selection algorithms. EnRank achieved the best AUC values using three out of the five classifiers. The Lasso-recommended features achieved the best AUC = 0.9946 while the EnRank-recommended features achieved the second best AUC = 0.9894. EnRank achieved the second best AUC = 0.8283 using the classifier DT, while Ridge-recommended features achieved the slightly better AUC = 0.8790.

The original version of EnRank integrated four feature selection algorithms Ttest/Chi2/Lasso/Ridge, which was compared with the new version integrating four new feature selection algorithms, as shown in Figure 9B. The four new feature selection algorithms were Variance Threshold (Variance), Mutual Information (MutualInfo), Extra Trees (ExtraTree), and ANOVA (Anova). The same model training and testing setting were carried out. The original version of EnRank outperformed the new version for all five classifiers. The best classifier LR was even improved by 0.0302 in the parameter-independent performance metric AUC.

The EnRank’s performance relied on the including efficient feature selection algorithms (Figure 9C). So a comparison was carried out for the performances of different combinations of feature selection algorithms. Here, we investigated the combinations of three or five algorithms. Figure 9C showed that the original version of EnRank achieved the best AUC value = 0.9446, although a slightly worse AUC = 0.9441 was achieved by removing Ttest.

### Biological Involvement of the EnRank-Detected Biomarkers

Table 3 listed the 50 EnRank-detected biomarkers and their corresponding gene information. Many transcriptomic biomarkers are from chromosomes 19 and 2. And two biomarkers ILMN_1807491 and ILMN_2323933 are from the same gene Leukocyte Associated Immunoglobulin Like Receptor 2 (LAIR2). Limited knowledge was known about the roles of LAIR2 in the PH patients, based on the information from PubMed (Fiorini et al., 2017) and MalaCards (Rappaport et al., 2017). There were five transcriptomic biomarkers with unknown chromosomal locations.

**Table 3:** Detailed information of the 50 EnRank-detected biomarkers.

Rank	Feature	Gene	Chr	Strand	Rank	Feature	Gene	Chr	Strand
41	ILMN_1804350	LOC644852	1	+	39	ILMN_1711786	NFE2	12	−
1	ILMN_1806023	JUN	1	**−**	20	ILMN_2207291	IFNG	12	**−**
15	ILMN_1723912	IFI44L	1	+	25	ILMN_2388547	EPSTI1	13	**−**
43	ILMN_2339955	NR4A2	2	**−**	40	ILMN_2229649	KCTD12	13	**−**
11	ILMN_1782305	NR4A2	2	**−**	5	ILMN_2058782	IFI27	14	+
23	ILMN_1800602	GCA	2	+	38	ILMN_1763364	WHDC1	15	+
8	ILMN_1733998	DHRS9	2	+	10	ILMN_2057836	RNU2	17|NT_113932.1	**−**
44	ILMN_1755643	MGAT4A	2	**−**	12	ILMN_1772796	DYNLL2	17	+
22	ILMN_1801307	TNFSF10	3	**−**	47	ILMN_1742618	XAF1	17	+
3	ILMN_2088437	CX3CR1	3	**−**	26	ILMN_1749722	RNF213	17	+
7	ILMN_1745788	CX3CR1	3	**−**	24	ILMN_2413331	TMEM107	17	**−**
33	ILMN_1801216	S100P	4	+	18	ILMN_1775304	DNAJB1	19	**−**
48	ILMN_1745522	PF4V1	4	+	46	ILMN_2302757	FCGBP	19	**−**
49	ILMN_1710734	GZMK	5	+	16	ILMN_1751607	FOSB	19	+
42	ILMN_1779147	ENC1	5	**−**	6	ILMN_1740875	FPR2	19	+
9	ILMN_1702691	TNFAIP3	6	+	28	ILMN_1807491	LAIR2	19	+
32	ILMN_1721113	HLA-C	6	**−**	45	ILMN_2323933	LAIR2	19	+
2	ILMN_1789074	HSPA1A	6	+	17	ILMN_1664861	ID1	20	+
34	ILMN_1697499	HLA-DRB5	6	**−**	29	ILMN_2083066	IGLL3	22	+
4	ILMN_1748473	GIMAP4	7	+	35	ILMN_1796830	UBE2L3	22	+
14	ILMN_1799467	SAMD9L	7	**−**	13	ILMN_1852793	UniGene|BC067908		
21	ILMN_1684982	PDK4	7	**−**	27	ILMN_1781236	RefSeq|XR_001116.1		
37	ILMN_1716733	MYOM2	8	+	30	ILMN_1678859	RefSeq|XM_938277.1		
50	ILMN_1773313	USMG5	10	**−**	31	ILMN_2165753	RefSeq|NM_001080840.1		
19	ILMN_1674063	OAS2	12	+	36	ILMN_1822671	UniGene|BC020840		

Column “Gene” gave the gene symbol for each biomarker. Some biomarkers may not reside in a protein-coding gene, and they may have no annotated gene information.

The feature ILMN_2088437 was from the gene C-X3-C Motif Chemokine Receptor 1 (CX3CR1), which was known to be involved in HIV proliferation (Mhandire et al., 2014; Guo et al., 2020). The absence of CX3CR1 was observed to provide protection from tissue destruction from chronic obstructive pulmonary disease (COPD; Lee, 2012). And the gene CX3CR1 also demonstrated differential expressions in the COPD patients (Huang et al., 2019). Another feature ILMN_1740875 was within the gene Formyl Peptide Receptor 2 (FPR2) encoded on chromosome 19, which was actively involved in the mononuclear phagocyte responses in Alzheimer disease (Iribarren et al., 2005). FPR2 also demonstrated its capability of promoting the chemotaxis and survival of neutrophils in the COPD patients (Iribarren et al., 2005).

The EnRank-recommended genes were analyzed using the online tool DAVID version 6.8 (Jiao et al., 2012). The list of genes was annotated to cover the top 50 EnRank-recommended features and was screened against the human genome. The statistical significance *p* values were adjusted by the multi-test Benjamini corrections, and only the functional terms with the Benjamini-corrected values of *p* < 0.05 were kept for further analysis. It is interesting to observe that no GO terms were significantly enriched in PH biomarkers; while seven KEGG pathways were enriched with PH biomarkers. Many of these KEGG pathways were associated with antiviral immunity. The most significant KEGG pathway was hsa05164 (Influenza A) with the Benjamini-corrected *p* value = 2.80e-4. The infection of influenza A caused a patient’s death after 3 months of treatment with the popular drug bosentan for pulmonary hypertension in a clinical trial (Hoeper et al., 2005). As of now, no direct link was presented in the literature. But virus infection is known to be closely connected with pulmonary hypertension (Kimura et al., 2019; Miyasaka et al., 2020; Table 4).

**Table 4:** Enriched functional terms of the 50 EnRank-detected PH biomarkers.

KEGG	Term	*p* values	Benjamini
hsa05164	Influenza A	3.50E-06	2.80E-04
hsa05162	Measles	3.30E-04	1.30E-02
hsa04612	Antigen processing and presentation	9.40E-04	2.20E-02
hsa05168	Herpes simplex infection	1.10E-03	2.20E-02
hsa05332	Graft-versus-host disease	3.30E-03	4.70E-02
hsa05169	Epstein-Barr virus infection	3.70E-03	4.70E-02
hsa05330	Allograft rejection	4.10E-03	4.70E-02

The screening was carried out using the online tool DAVID in the Gene Ontology (GO) terms and KEGG terms. The first two columns gave the KEGG IDs and the corresponding terms. The last two columns gave the statistical significance *p* values and the Benjamini-corrected *p* values. Only the terms with the Benjamini-corrected p values < 0.05 were kept for further analysis.

## Conclusion

This study proposed a novel ensemble filter feature selection algorithm EnRank by the weighted integration of four popular filter algorithms. Five classification algorithms were used to evaluate the filter algorithms. The EnRank-detected biomarkers demonstrated very good performances on the PH prediction problem. And most of these biomarkers also demonstrated close connections with the disease PH from the literature.

The proposed algorithm EnRank is a feature selection framework, and may integrate feature selection algorithms with feature weights. The main limitation of EnRank is the choices of feature selection algorithms to be integrated. The parameter pTopK may also impact the final model performances. Others may want to carry out a series of comparable experiments to find the best parameters for their own datasets.

## Data Availability Statement

Publicly available datasets were analyzed in this study. This data can be found here: https://www.ncbi.nlm.nih.gov/geo/query/acc.cgi?acc=GSE33463 [The dataset has the accession GSE33463 in the Gene Expression Omnibus (GEO) database] and https://www.ncbi.nlm.nih.gov/geo/query/acc.cgi?acc=GSE22356 [The dataset has the accession GSE22356 in the Gene Expression Omnibus (GEO) database].

## Author Contributions

FZ, XL, and RZ designed the project, carried out the experiments, and drafted the manuscript. XL, YZ, and CF were involved in the clinical annotations and results discussion. RZ carried out the coding of the computational analysis. RZ and FZ revised and polished the manuscript. All authors contributed to the article and approved the submitted version.

### Conflict of Interest

The authors declare that the research was conducted in the absence of any commercial or financial relationships that could be construed as a potential conflict of interest.
